# Malondialdehyde mediates oxidized LDL-induced coronary toxicity through the Akt-FGF2 pathway via DNA methylation

**DOI:** 10.1186/1423-0127-21-11

**Published:** 2014-02-03

**Authors:** Tzu-Ching Yang, Yi-Jie Chen, Shwu-Fen Chang, Chu-Huang Chen, Po-Yuan Chang, Shao-Chun Lu

**Affiliations:** 1 Department of Biochemistry and Molecular Biology, National Taiwan University College of Medicine, Taipei, Taiwan; 2 Graduate Institute of Medical Sciences, College of Medicine, Taipei Medical University, Taipei, Taiwan; 3 Department of Medicine, Vascular and Medicinal Research, Texas Heart Institute, Houston, TX, USA; 4 L5 Research Center, China Medical University Hospital, Taichung, Taiwan; 5 Center for Lipid Biosciences and Department of Medicine, Kaohsiung Medical University, Kaohsiung, Taiwan; 6 Cardiovascular Center and Division of Cardiology, Department of Internal Medicine, National Taiwan University Hospital and College of Medicine, No. 7, Chung-Shan South Road, Taipei 100, Taiwan

**Keywords:** DNA methylation, Epigenetics, Gene expression, Lipid oxidation, Lipoproteins, Malondialdehyde (MDA), Signal transduction

## Abstract

**Background:**

Oxidized LDL (oxLDL) is involved in the development of atherosclerotic heart disease through a mechanism that is not fully understood. In this study, we examined the role of malondialdehyde (MDA), an important oxidative stress epitope of oxLDL, in mediating coronary endothelial cytotoxicity.

**Results:**

Human coronary artery endothelial cells (HCAECs) were treated with oxLDL in the presence or absence of antibody against MDA (anti-MDA) or apoB100 (anti-apoB100). In HCAECs treated with oxLDL (100 μg/ml) alone, DNA synthesis, cell viability, and expression of prosurvival fibroblast growth factor 2 (FGF2) were significantly reduced (*P* < 0.01 vs phosphate buffered saline–treated cells). These inhibitory effects of oxLDL were significantly attenuated in HCAECs cotreated with anti-MDA (0.15 μg/ml; *P* < 0.05 vs oxLDL-treated cells), but not in those cotreated with anti-apoB100. When we tested the effects of a panel of signal transduction modifiers on the signal transduction pathways of MDA in oxLDL-treated HCAECs, we found that MDA-induced cytotoxicity was mediated partly through the Akt pathway. Using a reporter gene assay, we identified an oxLDL-response element in the *FGF2* promoter that was responsible for the transcriptional repression of *FGF2* by oxLDL. The results of bisulfite genomic DNA sequencing showed that in HCAECs treated with oxLDL, the GC-rich promoter of *FGF2* was heavily methylated at cytosine residues, whereas cotreatment with anti-MDA markedly reduced oxLDL-induced *FGF2* promoter methylation.

**Conclusion:**

OxLDL disrupts the growth and survival of HCAECs through an MDA-dependent pathway involving methylation of the *FGF2* promoter and repression of *FGF2* transcription. This novel epigenetic mechanism of oxLDL may underlie its atherogenicity in patients with atherosclerotic cardiovascular disease.

## Background

Oxidized low-density lipoprotein (oxLDL) has been shown to accumulate in atherosclerotic lesions, and a growing body of evidence indicates that oxLDL is involved in the pathogenesis of coronary artery disease, acute coronary syndrome, and vulnerable plaque [1]. Experimentally, oxLDL exposure induces a variety of effects in endothelial cells (ECs), such as the release of chemokines, cytokines, and growth factors; the expression of surface molecules that regulate endothelial permeability and hemostatic properties; and changes in cell growth, division, and death [2,3]. The pathophysiologic effects of oxLDL observed in vitro have been implicated in the initiation and progression of atherosclerosis in vivo [4].

Malondialdehyde (MDA) is an end-product of the radical-initiated oxidative decomposition of polyunsaturated fatty acids; therefore, it is frequently used as a biomarker of oxidative stress [5]. Clinical data have indicated that MDA-type epitopes are prominent and prevalent and are important in cardiovascular disease; thus, these antigens are key candidates for use in characterizing immune responses that are relevant to atherosclerosis [6,7]. The Veterans Affairs Diabetes Trial, a recent clinical trial that investigated the association between MDA and diabetes mellitus, showed that the levels of MDA-LDL in circulating immune complexes can predict the occurrence of myocardial infarction and acute cardiovascular events in patients with type 2 diabetes [8]. However, the precise mechanism by which MDA might be causing endothelial cytotoxicity is not known.

OxLDL has suppressive effects on genes that regulate endothelial function, such as nitric oxide synthase [9]. Furthermore, oxLDL can modulate the signal transduction pathway of receptor Gi coupling by downregulating the expression of heterotrimeric G protein Gαi2 [10]. In addition, we have previously shown that the endothelial damage caused by oxLDL is accompanied by the selective downregulation of fibroblast growth factor 2 (FGF2) and can be prevented by supplementing cells with exogenous FGF2 [11,12]. Using an in vitro model of angiogenesis, we also showed that capillary-like microtube growth was impaired in arterial explants from hypercholesterolemic rabbits or in those from normocholesterolemic rabbits exposed to oxLDL in vitro and that this reduced microtube growth was associated with reduced FGF2 concentrations in the culture medium of the explants [13]. The downregulation of FGF2 expression, therefore, has become a recognized mechanism of endothelial dysfunction.

FGF2 is a pleiotropic protein that regulates a wide range of functions and has angiogenic and anti-apoptotic effects on vascular ECs [14,15]. The reduction of FGF2 levels in ECs has detrimental effects, such as massive apoptosis and greatly impaired angiogenic responses [13]. When ECs are treated with homocysteine, FGF2 is downregulated at the transcriptional level by a mechanism involving DNA methylation of the GC-rich *FGF2* promoter [16]. However, how oxLDL and its oxidative stress epitopes, such as MDA, regulate transcription of *FGF2* is not known. In this study, we examined the role of MDA in mediating coronary endothelial cytotoxicity and addressed the question of whether oxLDL downregulates endothelial FGF2 via a signaling pathway that involves DNA methylation. We have identified a mechanistic model of EC gene modulation influenced by oxLDL and MDA epitopes.

## Methods

### Cells and preparation of LDL

Human coronary artery ECs (HCAECs, Clonetics, USA) were maintained from passages 4 to 7 in microvascular endothelial cell growth medium (EGM-MV) supplemented with 20% fetal bovine serum and antibiotics (100 μg/ml streptomycin, 100 IU/ml penicillin, and 0.25 μg/ml amphotericin B). Oxidized LDL was prepared as previously described [11,12], and precautions were taken to prevent endotoxin contamination. The protein concentration of each LDL preparation was determined by using the Lowry method, and thiobarbituric acid–reactive substances (TBARS) were determined as a measure of oxidative lipid modification [11,12].

### DNA synthesis analysis, cell counting, and enzyme-linked immunosorbent assay (ELISA)

For the DNA synthesis and intracellular FGF2 protein assays, HCAECs (1 × 10^6^) were seeded in each well of 12-well Corning cell culture plates (Corning, USA). HCAECs were incubated for 24 hours with or without oxLDL (100 μg/ml) in the presence of phosphate-buffered saline (PBS), goat polyclonal anti-MDA (0.01, 0.05, 0.10, or 0.15 mg/ml), anti-apoB100 (0.15 μg/ml) (both antibodies from Academy Bio-Medical Co., USA) [17], preimmune goat serum, or recombinant soluble human FGF2 (50 ng/ml; Upstate Biotechnology, USA). DNA synthesis was quantified by measuring ^3^H-thymidine incorporation, as previously described [11,12]. ^3^H-thymidine was from Moravek Biochemicals, Inc. (USA) or DuPont NEN (USA). Cells were viewed under an inverted microscope and were counted by using a hemocytometer. The percentage of dead cells was determined according to trypan blue positivity. FGF2 concentrations were measured with an ELISA by using a Quantikine kit (R&D Systems, USA), as previously described [11].

### Reverse transcription-polymerase chain reaction (RT-PCR)

RT-PCR was performed with total RNA and PCR primers for *FGF2* or the gene encoding β-actin (used as an internal control), according to a previously described protocol [12]. The *FGF2* primers were as follows: 5′-GGA-GTG-TGT-GCT-AAC-CGT-TAC-CTG-GCT-ATG-3′ (upstream) and 5′-TCA-GCT-CTT-AGC-AGA-CAT-TGG-AAG-AAA-AAG-3′ (downstream). β-actin primers were as follows: 5′-AAC-CGC-GAG-AAG-ATG-ACC-CAG-ATC-ATG-TTT-3′ (upstream) and 5′-AGC-AGC-CGT-GGC-CAT-CTC-TTG-CTC-GAA-GTC-3′ (downstream). A fraction of each PCR product (10 μl) was analyzed by using gel electrophoresis (2% agarose), and DNA bands were stained with ethidium bromide and visualized by using ultraviolet transillumination. Densitometric quantification was performed by using a PhosphorImager (Molecular Dynamics, USA).

### Inhibitors of signal transduction pathways

To characterize the involvement of major signal transduction pathways, cells treated with or without anti-MDA were also treated with 100 ng/ml pertussis toxin (PTX, a G*i* protein inhibitor), 1 μg/ml Akt inhibitor (1 L6-hydroxymethyl-chiro-inositol-2-[R]-2-O-methyl-3-O-octadecyl-sn-glycerocarbonate), or 0.4 μg/ml 5-aza-deoxycytidine (5-aza-dC; a methylation inhibitor) for 24 hours before exposure to oxLDL (100 μg/ml). All agents were purchased from Calbiochem (USA). Protocols for individual agents were determined on the basis of the maximal doses and durations tolerable by the cells; tolerability was defined as <10% reduction in cell viability, as determined by using the MTT assay.

### Cell viability MTT assay

HCAECs (5 × 10^4^ cells/well) were dispensed into 24-well plates and incubated for 24 hours with oxLDL (100 μg/ml) in the presence or absence of different treatments, and the index of EC viability was determined by using the colorimetric tetrazolium (MTT) assay. Absorbance was measured at a wavelength of 540 nm for viable cells by using a microplate reader (Thermo Electron Corporation, USA).

### DNA constructs, cell transfection, and luciferase reporter gene assay

The reporter gene assay was performed by using a dual-luciferase expression system (Promega, USA). Human *FGF2* 5′-flanking sequences [18] were amplified from human genomic DNA by using PCR, cloned into the pGL3-basic firefly luciferase reporter vector (Promega, USA), and sequenced. HCAECs were grown to 80% confluency in plastic 12-well plates and then transfected with 0.75 μg of pGL3-basic or an equimolar amount of the different pGL3-*FGF2* constructs by using Superfect™ reagent, according to the manufacturer’s instructions (Qiagen, USA). Cotransfection with 0.5 μg of the *Renilla* luciferase expression vector phRL-TK was used as an internal control. Twenty-four hours after transfection, the cells were treated for 24 hours with 100 μg/ml oxLDL. Cell lysates were then prepared for luciferase assays by using Luciferin and a luminometer (Packard Instrument Company, Inc., USA). The promoter activity of the reporter construct was normalized to the promoter activity of phRL-TK and expressed as the fold increase relative to that in cells transfected with pGL3-basic.

### DNA sequence analysis and bisulfite genomic DNA sequencing

The 5′-flanking sequence of the human *FGF2* gene was retrieved from GenBank and analyzed for CpG islands by using “CpG Island Searcher,” available at the website http://www.cpgislands.com[19,20]. Transcription factor binding sites were analyzed by using the TRANSFAC database, available at http://www.gene-regulation.com/. To study DNA methylation, HCAECs that were cultured to 80% to 90% confluency were subjected to different treatments, followed by genomic DNA extraction performed according to standard procedures. With the use of the EpiTect bisulfite kit (Qiagen, USA), the reaction of genomic DNA (2 μg) with bisulfite converted all unmethylated cytosine residues to uracil. Bisulfite-modified DNA was amplified with *FGF2*-specific primers by using previously described cycling conditions [16].

### Statistical analysis

The significance of the differences between group means was assessed by using a 2-way Student *t* test for single comparisons and the Bonferroni test for multiple comparisons. Analysis of variance (ANOVA), followed by Scheffé’s test for significance was used to compare values for concentration- and time-dependent responses. Probability values <0.05 were considered significant. Results are expressed as the mean ± SEM.

## Results

### MDA mediates the effects of oxLDL on DNA synthesis, EC survival, and FGF2 expression

The exposure of HCAECs to oxLDL for 24 hours decreased DNA synthesis and cell viability in a concentration-dependent manner (Additional file 1: Figure S1). At a concentration of 100 μg/ml, oxLDL significantly reduced DNA synthesis and cell viability (*P* < 0.05 vs untreated control cells) without significantly increasing the percentage of dead cells (Additional file 1: Figure S1). At concentrations >100 μg/ml, oxLDL not only significantly decreased DNA synthesis and cell viability, but it also significantly increased the percentage of dead cells (*P* < 0.01; Additional file 1: Figure S1). Therefore, we used an oxLDL concentration of 100 μg/ml for all subsequent experiments.

**Figure 1:**
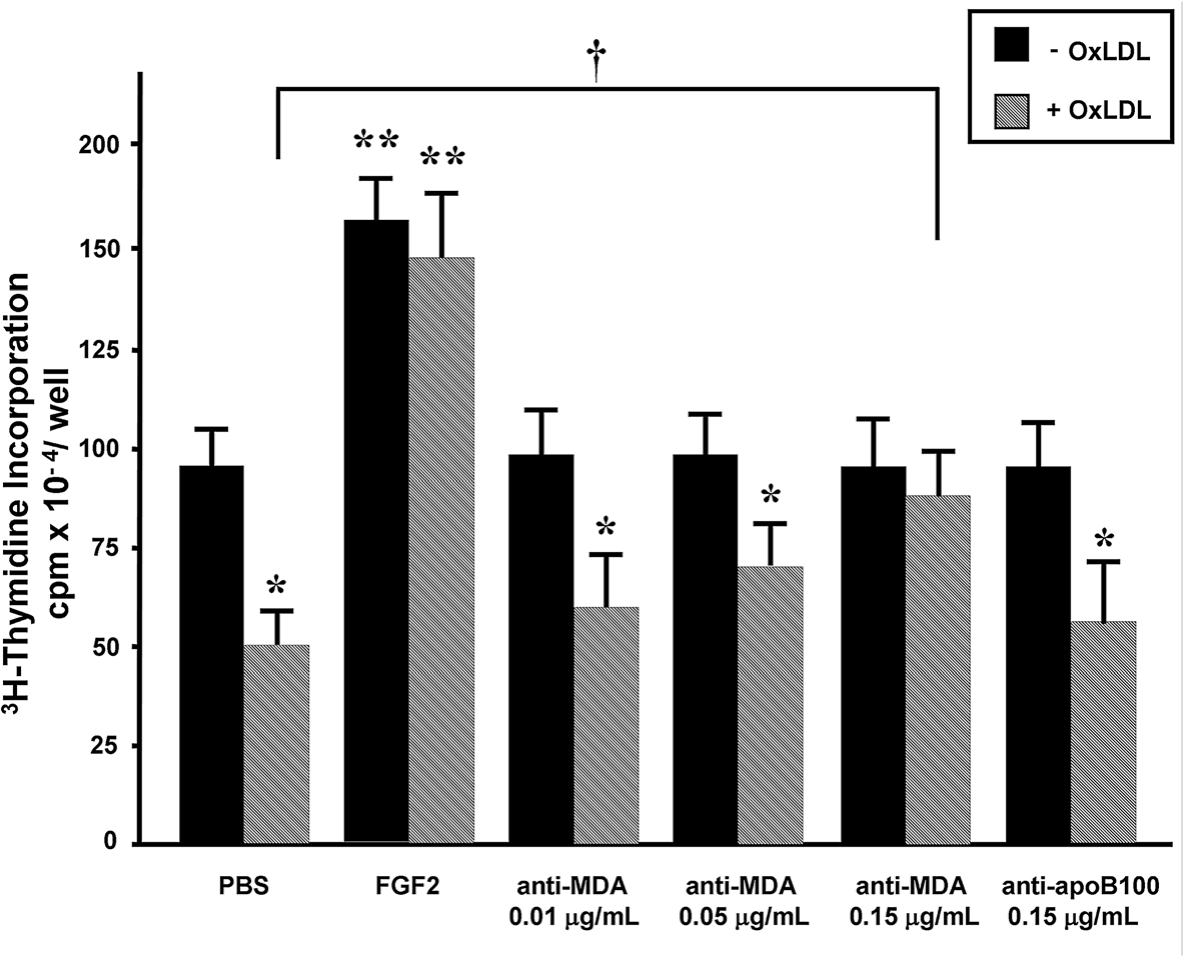
**Role of the MDA epitope on the oxLDL-induced reduction of DNA synthesis, as determined by measuring **^**3**^**H-thymidine incorporation.** Cultured human coronary artery endothelial cells were incubated for 24 hours with or without oxLDL (100 μg/ml) in the presence or absence of anti-MDA (0.01, 0.05, or 0.15 μg/ml), anti-apoB100 (0.15 μg/ml), or FGF2 (50 ng/ml). Values shown are the mean ± SEM (n = 3). ^*^P < 0.05, ^**^P < 0.01 vs the oxLDL-untreated, PBS-treated control (first black column)*. *^†^*P* < 0.05 between the indicated pair. MDA, malondialdehyde; oxLDL, oxidized low-density lipoprotein.

**Figure 2:**
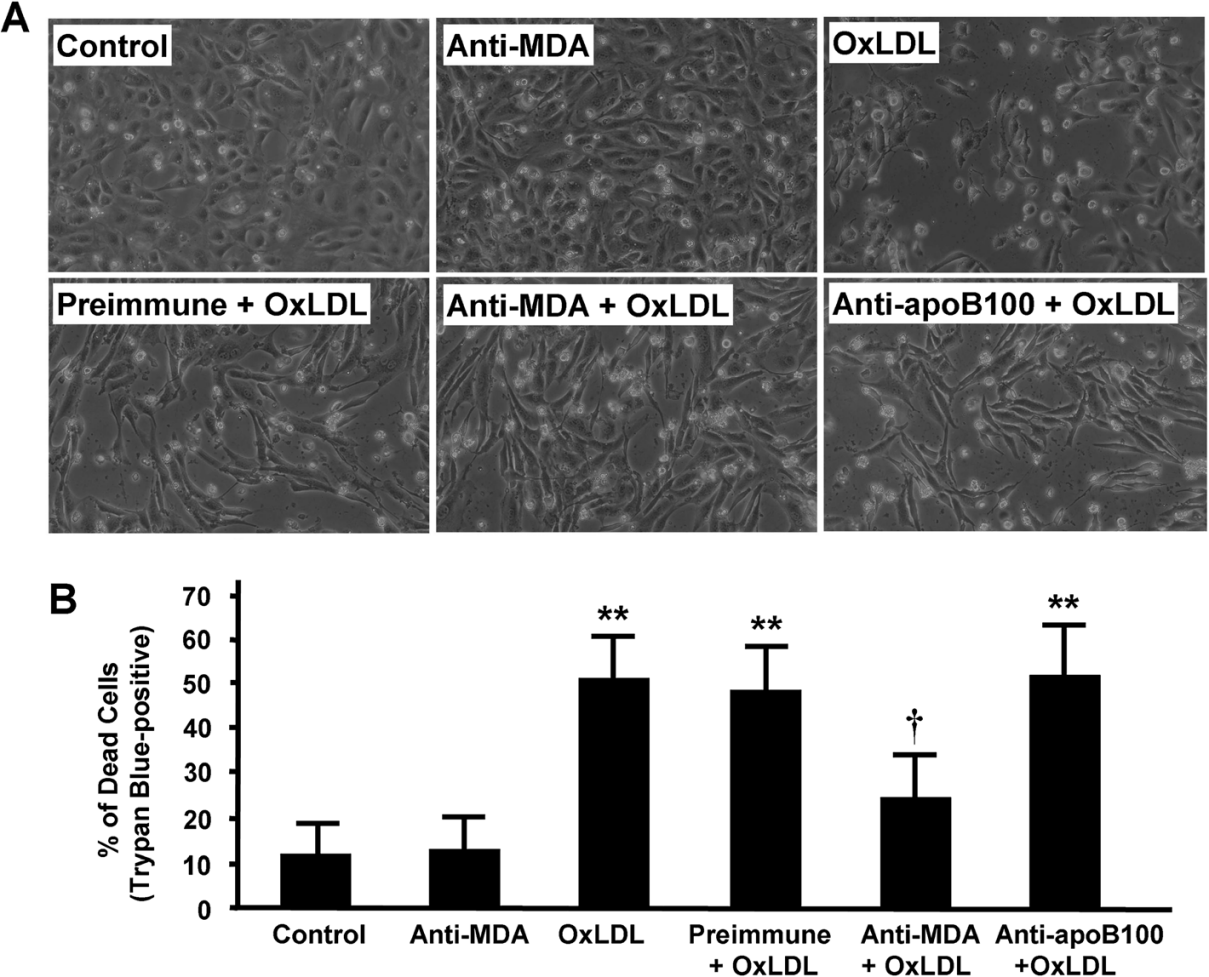
**Role of the MDA epitope in oxLDL-induced human coronary artery endothelial cell (HCAEC) cytotoxicity. (A)** HCAECs were incubated for 24 hours with or without oxLDL (100 μg/ml) in the presence or absence of anti-MDA (0.15 μg/ml), preimmune serum, or anti-apoB100 (0.15 μg/ml), as indicated; control cells were treated with PBS. Cells are shown under the view of an inverted microscope. **(B)** Percentage of trypan blue–positive cells expressed as the mean ± SEM (n = 3). **P < 0.01 vs control. ^**†**^*P* < 0.05 vs oxLDL alone. MDA, malondialdehyde; oxLDL, oxidized low-density lipoprotein.

After HCAECs were treated with oxLDL (100 μg/ml) for 24 hours, DNA synthesis was decreased by 40% to 50% (Figure 1), a finding similar to those reported previously [11,12]. Treatment of HCAECs with FGF2 (50 ng/ml) alone had a potent mitogenic effect (*P* < 0.05), and cotreatment with FGF2 and oxLDL prevented the oxLDL-mediated inhibition of DNA synthesis. The inhibitory effect of oxLDL on DNA synthesis was also markedly attenuated in HCAECs cotreated with 0.15 μg/ml anti-MDA, but not in those cotreated with anti-apoB100 (Figure 1). Treatment of HCAECs with oxLDL alone reduced the number of cells (Figure 2A) and significantly increased the percentage of trypan blue–positive cells (Figure 2B, *P* < 0.01 vs control), whereas treatment with anti-MDA alone (0.15 μg/ml) had a negligible effect. Cotreatment of HCAECs with anti-MDA and oxLDL attenuated the cytotoxic effects of oxLDL (Figure 2A and B, *P* < 0.05 vs oxLDL alone), whereas treatment with preimmune serum or anti-apoB100 had no protective effect (Figure 2A and B).

**Figure 3:**
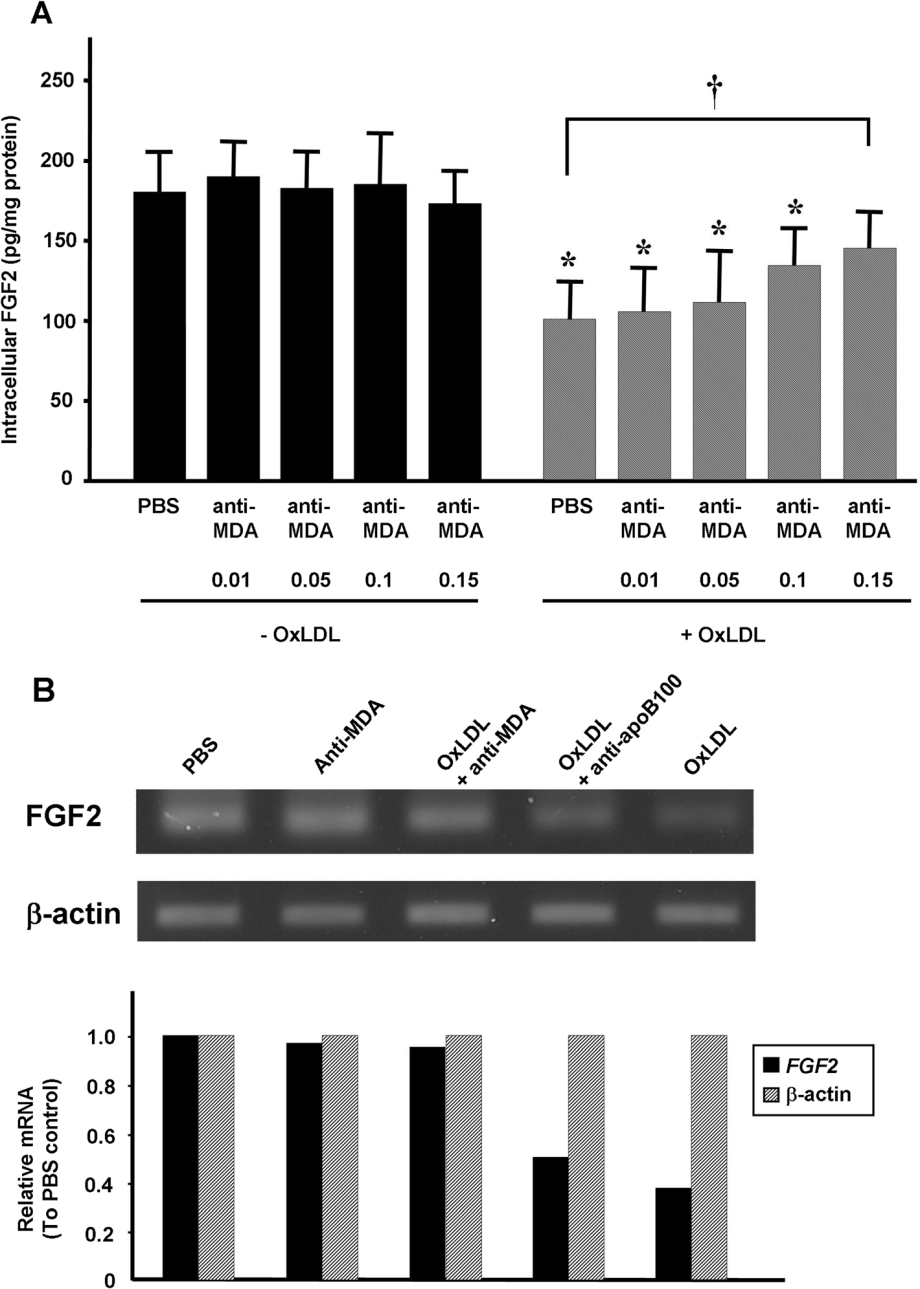
**Effect of anti-MDA on oxLDL-regulated FGF2 expression. (A)** Intracellular FGF2 protein concentrations in cultured human coronary artery endothelial cells (HCAECs) are shown, as determined with ELISA. Cells were incubated for 24 hours with or without oxLDL (100 μg/ml) in the presence or absence of anti-MDA (0.01, 0.05, 0.1, or 0.15 μg/ml). Values shown are the mean ± SEM (n = 3). *P < 0.05, **P < 0.01 vs the oxLDL-untreated PBS control. ^**†**^*P* < 0.05 vs the oxLDL-treated PBS control. **(B) ***FGF2* mRNA levels in HCAECs relative to those in the PBS-treated control, as determined by using RT-PCR. HCAECs were incubated for 24 hours with the indicated reagents, and total RNA was subjected to RT-PCR analysis by using specific primers for *FGF2* and the gene encoding β-actin (internal control). The upper panel shows a gel representative of 3 separate experiments, and the lower panel shows the densitometric quantification. MDA, malondialdehyde; oxLDL, oxidized low-density lipoprotein.

Because anti-MDA prevented the antiproliferative effect of oxLDL, we examined the effects of anti-MDA on FGF2 production by using ELISA. In HCAECs treated with oxLDL (100 μg/ml), the intracellular FGF2 concentration was decreased by 40% to 50% (Figure 3A, *P* < 0.05). In oxLDL-untreated HCAECs, FGF2 levels were similar between cells treated with PBS and those treated with anti-MDA (0.01-0.15 μg/ml); however, in oxLDL-treated cells, cotreatment with anti-MDA attenuated the inhibitory effect of oxLDL on FGF2 expression in a dose-dependent manner (Figure 3A, *P* < 0.05 vs PBS + oxLDL). In addition, RT-PCR analysis showed that oxLDL reduced *FGF2* mRNA levels by ~50% when compared to the PBS control—an effect that was prevented by anti-MDA (0.15 μg/ml) but not anti-apoB100 (0.15 μg/ml) (Figure 3B).

### The effects of MDA are mediated through Akt and involve DNA methylation

**Figure 4:**
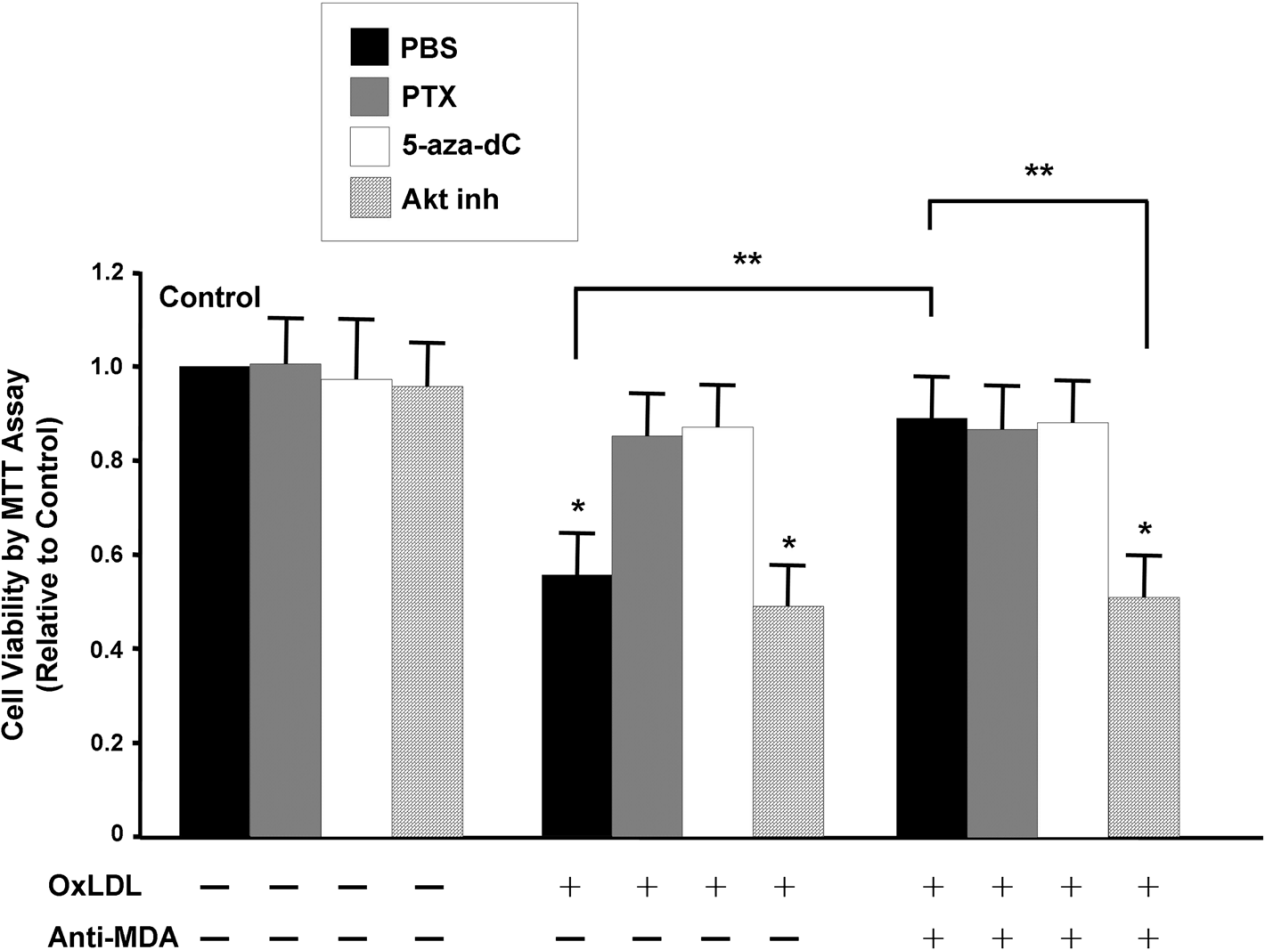
**Effects of signal transduction inhibitors on the oxLDL-induced reduction of cell viability, as determined by using the MTT assay in cultured human coronary artery endothelial cells (HCAECs).** HCAECs were incubated for 24 hours with signaling pathway modifiers in the presence or absence of oxLDL (100 μg/ml) and anti-MDA (0.15 μg/ml), as indicated. The cell viability after each treatment was normalized to that of the PBS control (first black column). Values shown are mean ± SEM (n = 3). *P < 0.05 vs control (first black column); **P < 0.05 between the indicated pair. 5-aza-dC, 5-aza-deoxycytidine; Akt inh, Akt inhibitor; MDA, malondialdehyde; oxLDL, oxidized low-density lipoprotein; PTX, pertussis toxin.

To investigate the signal transduction pathways of MDA in oxLDL-treated HCAECs, we examined the effects of pharmacologic inhibitors on cell viability (Figure 4). Compared with cells treated with PBS only (first black column), cells also treated with oxLDL (100 μg/ml) for 24 hours showed a 40% decrease in cell viability. This inhibitory effect of oxLDL was remarkably blocked in cells cotreated with either PTX, a G protein inhibitor, or with 5-aza-dC, a methylation inhibitor. When HCAECs were cotreated with both oxLDL and anti-MDA, the oxLDL-induced reduction in cell viability was blocked, which was unaffected by the addition of PTX or 5-aza-dC. However, the addition of Akt inhibitor significantly attenuated the effect of anti-MDA (last column). These results indicate that the Akt signaling pathway and DNA methylation are involved in mediating cell proliferation in anti-MDA/oxLDL-treated HCAECs.

### The *FGF2* promoter is regulated by oxLDL and MDA

**Figure 5:**
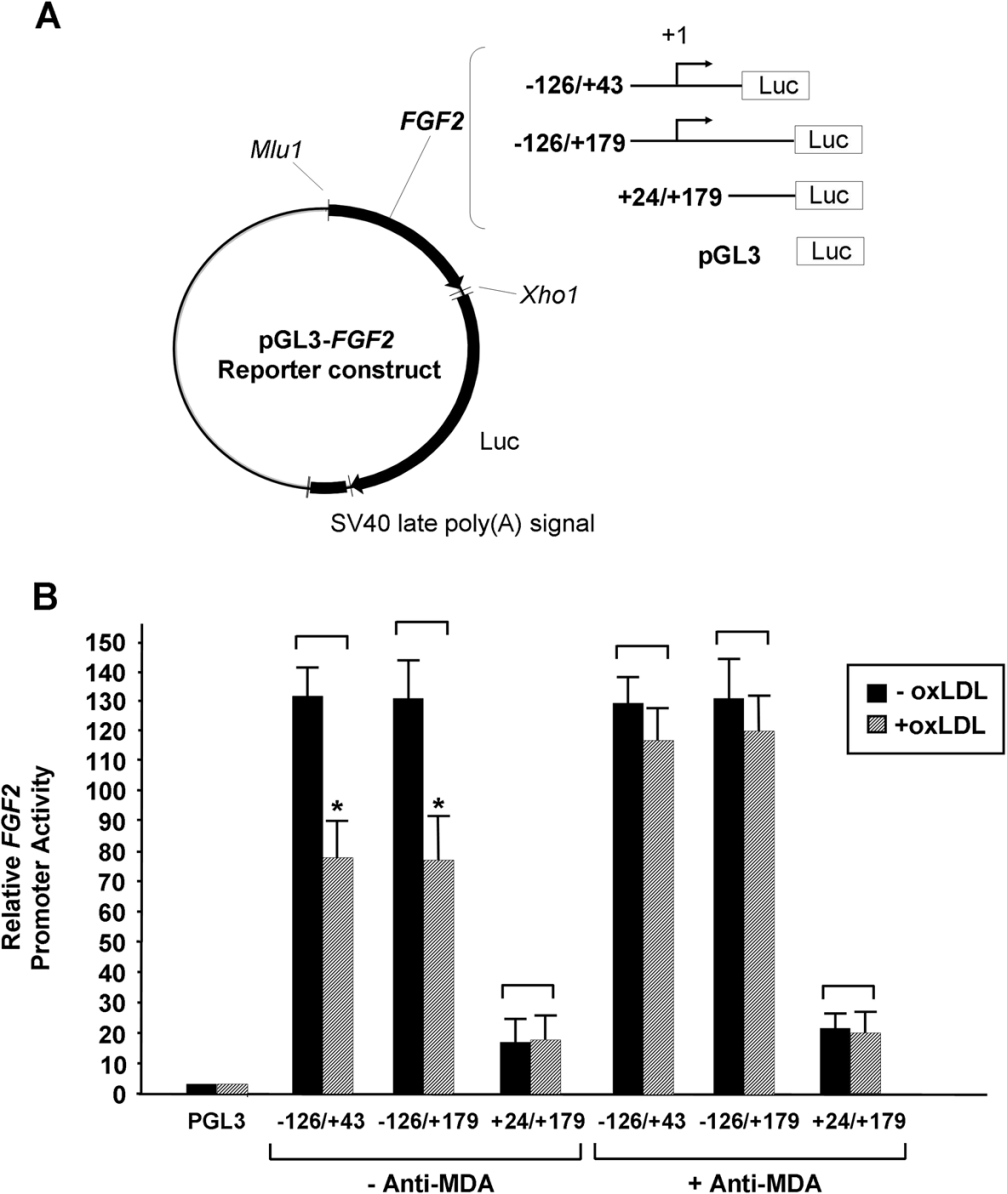
**Effects of anti-MDA and oxLDL on the transcriptional regulation of *FGF2 *in cultured human coronary artery endothelial cells (HCAECs). (A)** A diagram showing the different *FGF2* reporter gene constructs used. Different lengths of human *FGF2* 5′-flanking sequences were cloned into the pGL3-basic luciferase reporter vector. +1 indicates the transcription start site. **(B)***FGF2* promoter activity is shown, as determined by using a luciferase reporter gene assay. HCAECs were cotransfected with the indicated constructs and phRL-TK (internal control) and were incubated for 24 h with or without oxLDL (100 μg/ml) in the presence or absence of anti-MDA (0.15 μg/ml). Luciferase activities are expressed relative to those in cells transfected with pGL3-basic. Values shown are the mean ± SEM (n = 3-5). *P < 0.05 vs corresponding oxLDL-untreated controls. Luc, luciferase reporter gene; MDA, malondialdehyde; oxLDL, oxidized low-density lipoprotein.

To determine whether the reduced FGF2 expression observed in oxLDL-treated cells resulted from decreased *FGF2* gene transcription, we evaluated *FGF2* promoter activity in the presence or absence of oxLDL (100 μg/ml) by using a luciferase reporter gene assay. Human *FGF2* genomic sequences containing the *FGF2* promoter were generated by using PCR and were cloned into the firefly luciferase vector, pGL3 (Figure 5A). In the absence of oxLDL, the –126/+ 43 and –126/+179 constructs containing the *FGF2* promoter induced *FGF2* gene expression 120-fold more than did the pGL3-basic vector (Figure 5B). Deletion of the *FGF2* promoter from position –126 to + 24 resulted in an 80% reduction in *FGF2* gene expression, confirming the removal of a basal promoter in this region. The addition of oxLDL (100 μg/ml) reduced *FGF2* expression in cells transfected with constructs –126/+ 43 and –126/+179 by 30% to 40% (*P* < 0.05). However, the presence of anti-MDA prevented the oxLDL-induced repression of *FGF2* promoter activity. Furthermore, attenuation of the oxLDL-induced repression of *FGF2* promoter activity by anti-MDA was partially prevented by Akt inhibitor (Additional file 1: Figure S2), suggesting the involvement of Akt in the regulation of *FGF2* expression by MDA and oxLDL. Our results suggest that *FGF2* transcription in HCAECs can be repressed by oxLDL and that this effect is mediated through the MDA epitope. The preservation of *FGF2* promoter activity by anti-MDA is in agreement with our RT-PCR results (Figure 3B).

### OxLDL and MDA regulate the *FGF2* promoter via methylation of a CpG island

**Figure 6:**
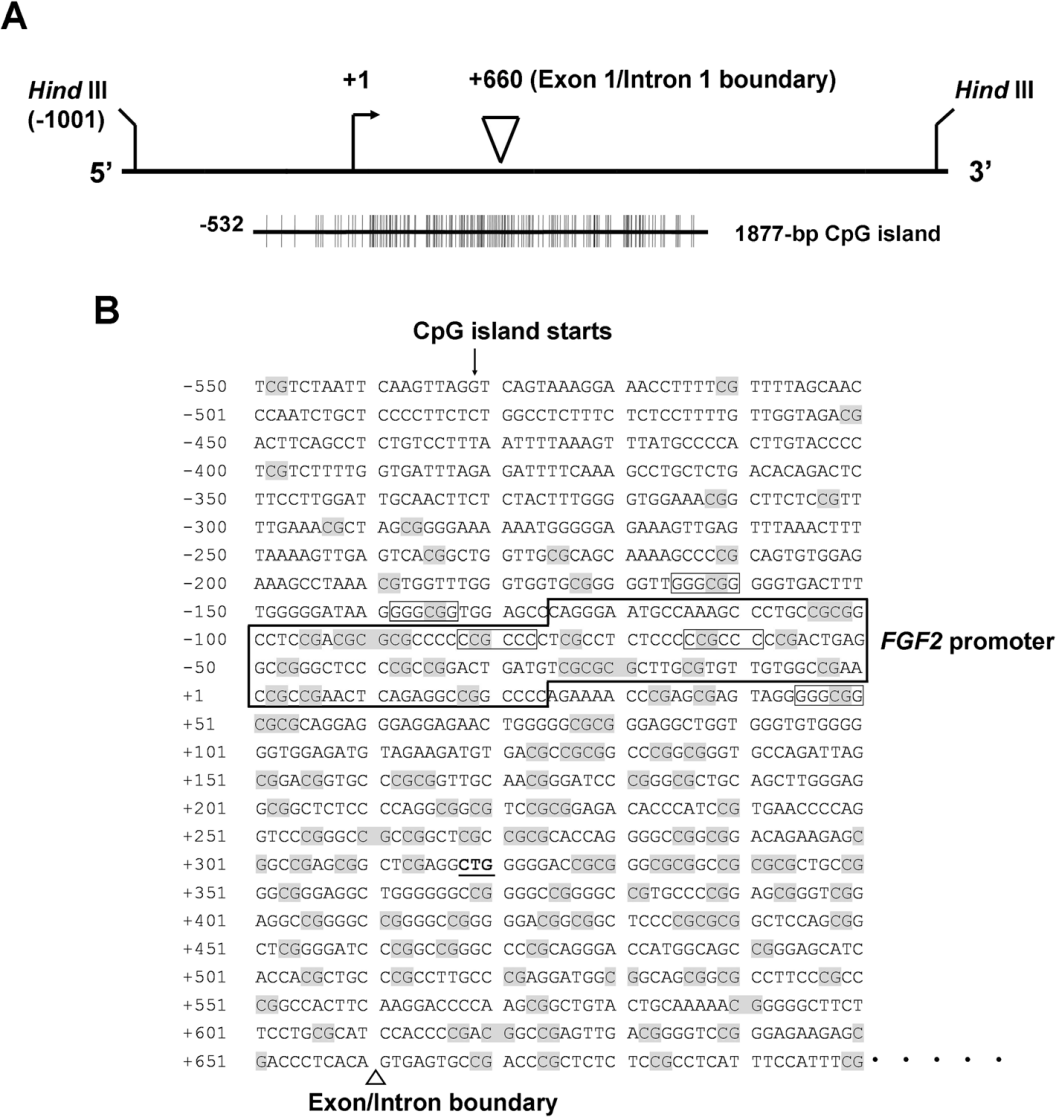
**The human *FGF2 *gene promoter. (A)** Schematic representation of the CpG island promoter of human *FGF2*. The numbers are the nucleotide positions relative to the +1 transcription start site. Each CpG dinucleotide is shown by a vertical line. **(B)** Part of the genomic DNA sequence of *FGF2* is shown, featuring the *FGF2* promoter, GC boxes (outlined), and CpG dinucleotides (shaded). The CpG island start site (downward arrow in **B**), the exon-intron boundary (triangle in **B**), and the translation start codon (**CTG** in **B**) are indicated.

Genomic sequence analysis of human *FGF2* confirmed the absence of a TATA sequence and revealed the presence of multiple GC boxes (GGGCGG or CCGCCC) (Figure 6). Using the “CpG Island Searcher” database to search for CpG dinucleotides, we identified a 1877-bp CpG island that starts at –532 in the 5′-flanking region of *FGF2* and extends through exon 1 into the first intron. This portion of the human *FGF2* gene contains the oxLDL-responsive promoter that we previously showed can be regulated by homocysteine [16]. A computer scan of the TRANSFAC database disclosed several possible binding sites for transcription factors, including 2 GC boxes in the oxLDL/MDA-responsive promoter (Figure 6).

**Figure 7:**
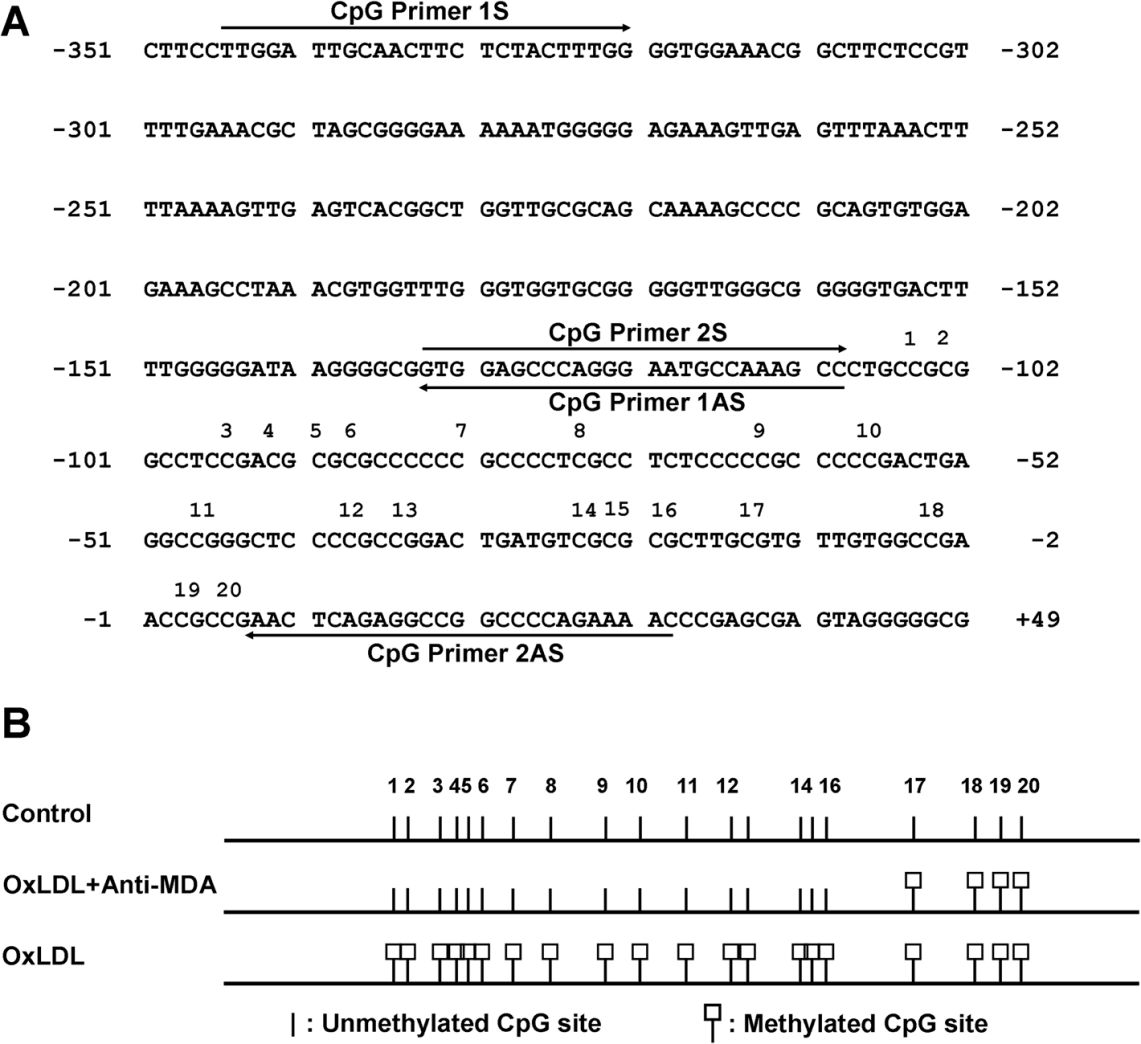
***FGF2 *promoter analysis. (A)** The CpG island sequence in the promoter region of human *FGF2* is shown. The human *FGF2* promoter contains 20 CpG sites, numbered 1-20. The primers used for DNA methylation sequencing are shown. **(B)** An illustration showing the methylation status of the CpG dinucleotides in human coronary artery endothelial cells treated for 24 hours with oxLDL (100 μg/ml) in the presence or absence of anti-MDA (0.15 μg/ml). Unmethylated cytosines are indicated by stalks. Methylated cytosines are indicated by stalks with square heads. MDA, malondialdehyde; oxLDL, oxidized low-density lipoprotein.

Because CpG methylation is a key factor for *FGF2* gene expression, we investigated whether MDA mediates the oxLDL-induced suppression of *FGF2* expression through this epigenetic modification. The methylation status of CpG dinucleotides in the *FGF2* promoter was characterized by performing bisulfite genomic DNA sequencing in HCAECs (Figure 7). Twenty CpG dinucleotides (numbered 1–20 in Figure 7) in the *FGF2* promoter region were analyzed by using 2 pairs of primers (CpG primer 1S/1AS; CpG primer 2S/2AS) designed to amplify the *FGF2* promoter region (Figure 7A). In PBS-treated control cells, none of the 20 cytosine residues was methylated (Figure 7B). In contrast, all 20 cytosine residues were methylated in cells treated with oxLDL (100 μg/ml). Furthermore, when anti-MDA was added to oxLDL-treated HCAECs, methylation of the cytosine residues was markedly reduced: only 4 of the 20 remained methylated (Figure 7B).

To determine whether oxLDL induced the upregulation of DNA methyltransferases in HCAECs, we examined the mRNA levels of DNA methyltransferase (*DNMT)1*, *DNMT3A*, and *DNMT3B* by using real-time PCR. We observed a modest oxLDL-induced increase in the levels of *DNMT1* and *DNMT3B* mRNA, which was attenuated by anti-MDA (Additional file 1: Figure S3). These data indicate that oxLDL represses *FGF2* transcription in ECs by promoting the methylation of CpG dinucleotides in the *FGF2* promoter—an action that is mediated through the oxidative MDA epitope of oxLDL.

## Discussion

**Figure 8:**
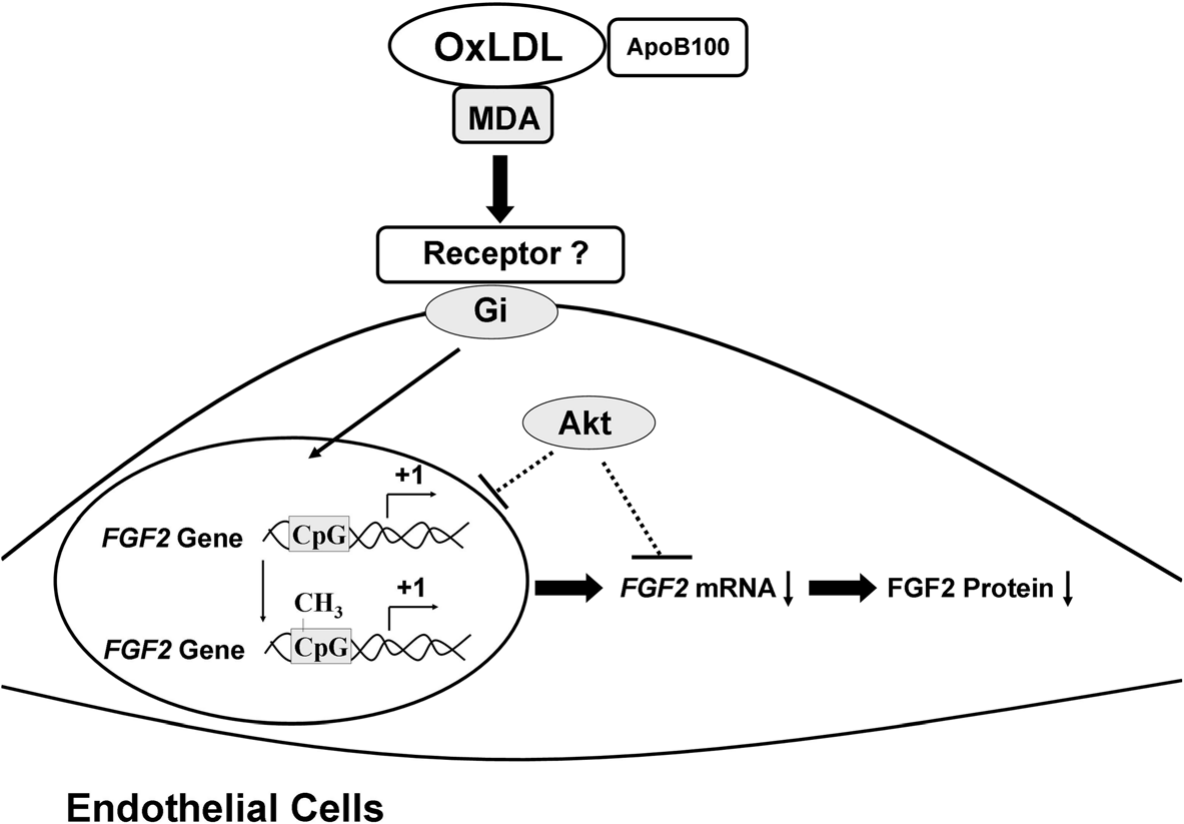
**A schematic illustration of FGF2 regulation by the MDA epitope of oxLDL in human coronary artery endothelial cells.** The transcription start site of the *FGF2* gene is indicated as +1. Methylation of the cytosine residues of CpG dinucleotides is indicated by –CH_3_. Arrows indicate stimulation or direction of transportation. Lines with an end bar indicate inhibition.

The aim of this study was to elucidate the mechanisms underlying the regulation of cell proliferation and FGF2 expression in ECs exposed to oxLDL. We found that oxLDL-induced EC damage can be prevented by anti-MDA, which blocks the oxLDL-induced reduction of intracellular FGF2 levels through an Akt-dependent pathway. Furthermore, the effect of MDA was mediated through the transcriptional repression of the *FGF2* promoter, which involved CpG methylation. Thus, we showed that MDA exerts its deleterious effects on ECs through an FGF2-dependent pathway, which may be the mechanism underlying the atherogenic nature of oxLDL. The schematic in Figure 8 outlines the signaling pathway through which MDA mediates endothelial cytotoxicity in the presence of oxLDL.

FGF2, a potent angiogenic factor involved in all aspects of angiogenesis (eg, EC proliferation and migration and vascular differentiation), is an indicator of EC survival [21,22]. Previously, we showed that both oxLDL and electronegative LDL downregulate endothelial FGF2 expression by inhibiting Gi, a heterotrimeric G protein [11,12]. In the current study, PTX, a Gi inhibitor, attenuated oxLDL-mediated effects on FGF2 expression, further supporting the important role of Gi protein in the oxLDL signaling pathway. Because circulating oxLDL and MDA-LDL levels are greatly increased in individuals who have coronary events [5-8,23], FGF2 downregulation by oxLDL may be an important mechanism underlying the impaired angiogenic response in atherosclerosis. In addition, involvement of the MDA epitope in oxLDL-induced angiostatic effects supports the use of MDA as a biomarker in cardiovascular diseases [5-8].

Although oxLDL is known to modulate the expression of many genes, its effect on *FGF2* promoter activity has not been previously reported. We found that the oxLDL-induced downregulation of FGF2 expression is caused by transcriptional repression of the *FGF2* gene promoter and that this repression could be effectively prevented by 5-aza-dC. The 5′-flanking regions of the *FGF2* promoter are responsible for the basal transcription of the *FGF2* gene, the subcellular distribution of FGF2 protein in adrenal medullary cells [18,24], and the cell density–dependent regulation of FGF2 levels in human astrocytes [25]. In the current study, we identified a 5′-flanking region in the *FGF2* promoter from –126 to +24 (which conforms to the published mapping of the *FGF2* gene promoter) that functions as an oxLDL-responsive element. The *FGF2* promoter is located in a CpG island, which is defined as a region of DNA larger than 500 bp that has a moving average %(G + C) greater than 55 and an observed/expected CpG dinucleotide ratio greater than 0.65 [19,20]. The CpG island of the human *FGF2* gene contains multiple GC boxes (GGGCGG or CCGCCC) and a basal promoter, which responded to oxLDL in our transient transfection system. We found that the CpG-rich *FGF2* promoter was heavily methylated by oxLDL, resulting in the subsequent suppression of *FGF2* transcription.

Previously, we have shown that L5, the most electronegative subfraction of LDL obtained by using anion-exchange chromatography, downregulates endothelial FGF2 expression at the transcriptional level by inhibiting Akt phosphorylation [26]. Furthermore, it has been shown that the L5-induced reduction in FGF2 expression can be attenuated by aspirin through a signaling pathway that requires Akt [27]. In the present study, the ability of Akt inhibitor to counteract the effects of anti-MDA in HCAECs further supports the important role of Akt in the oxLDL-FGF2 signaling pathway. Moreover, the involvement of Akt in the regulation of the *FGF2* promoter by oxLDL also implies a complex interplay between cellular survival and different signaling pathways in the response to atherogenic oxLDL.

Epigenetics is a phenomenon characterized by heritable changes in the phenotypic expression of genetic information that occurs without changes in DNA sequence. Emerging data suggest that epigenetic modifications affect the development of cardiovascular diseases, such as human heart failure and atherosclerosis [28,29]. DNA methylation, an epigenetic modification that occurs throughout the genome, is the addition of a methyl group to the cytosine residues preceding a guanosine (ie, CpG dinucleotides). This modification has been studied extensively and represents a well-understood epigenetic mechanism. Abnormal methylation of CpG islands in the promoter region of genes can be caused by exogenous stimuli such as smoking and oxidative stress, often leading to the silencing of genetic information and subsequent alterations in biologic function [30].

OxLDL has been shown to alter DNA promoter methylation in cultured ECs. In human umbilical vein ECs (HUVECs), oxLDL-induced apoptosis has been associated with upregulation of the proapoptotic genes *LOX-1*, *ANXA5*, *BAX*, and *CASP3* and inhibition of the anti-apoptotic genes *BCL2* and *cIAP-1*. Interestingly, the upregulation of these apoptosis-related genes was consistently accompanied by reciprocal changes in the methylation of the respective promoter regions [31]. However, promoter methylation induced by oxLDL is not always accompanied by reciprocal changes in gene expression. At different concentrations, oxLDL can differentially regulate the methylation status of the estrogen receptor alpha gene [32]. In addition, oxLDL can upregulate microRNA-29b, leading to epigenetic modifications of the matrix metalloproteinase (MMP)-2/MMP-9 genes [33]. Our discovery that the oxLDL-induced downregulation of FGF2 expression can be attenuated by 5-aza-dC, a well-known demethylating reagent [34], also supports that oxLDL promotes hypermethylation of the *FGF2* promoter, as illustrated in Figure 8.

Our study shows that oxLDL can specifically downregulate FGF2 expression in arterial ECs, revealing a novel, angiostatic function for oxLDL. Because oxLDL and homocysteine are both risk factors for atherosclerosis, the possibility remains that they synergistically promote EC cytotoxicity through a shared pathway related to FGF2 [16]. The notion that there is close interplay between homocysteine and oxLDL has been suggested by previous findings showing that homocysteine enhances the oxidation of human LDL in HUVECs and facilitates the subsequent uptake of oxLDL by macrophages [35]. Moreover, homocysteine can upregulate the expression of lectin-like oxLDL receptor-1 (LOX-1) in endothelial and mononuclear cells and increase the toxicity of oxLDL [36]. Collectively, our results support the idea that cross-talk occurs between the cardiovascular risk factors oxLDL and homocysteine and that the upregulation or reintroduction of FGF2 may counteract their pathologic effects in ECs.

## Conclusions

We have shown that oxLDL-induced impairment of EC integrity and survival is mediated by the MDA epitope and involves *FGF2* promoter methylation and the downregulation of intracellular FGF2. This signaling pathway through which MDA mediates endothelial cytotoxicity in the presence of oxLDL is outlined in Figure 8. Because high circulating levels of oxLDL and MDA-LDL are strongly associated with atherosclerotic diseases [37,38], we propose that the maintenance of FGF2 expression may provide protection of the endothelium in patients with these diseases. Furthermore, DNA demethylation may have therapeutic applications for protecting ECs against oxLDL-induced cell death and for preventing plaque instability.

## Abbreviations

5-aza-dC: 5-aza-deoxycytidine; ApoB100: Apolipoprotein B100; HCAEC: Human coronary artery endothelial cell; EC: Endothelial cell; FGF2: Fibroblast growth factor 2; MDA: Malondialdehyde; oxLDL: Oxidized low-density lipoprotein; PTX: Pertussis toxin.

## Competing interests

The authors declare that they have no competing interests.

## Authors’ contributions

PYC and SCL conceived and designed the experiments. TCY and YJC performed the experiments. PYC, SFC, and CHC analyzed the data. TCY, PYC, LSC and CHC wrote the paper. All authors have read and approved the final manuscript.

## Supplementary Material

Additional file 1: Figure S1 Effects of oxLDL on DNA synthesis, cell viability, and cell death in cultured HCAECs. Cells were treated with increasing concentrations of oxLDL for 24 hours, and DNA synthesis (•), cell viability (□), and cell death (Δ) were assessed. Values are expressed as the mean±SEM (n=3). *P < 0.05, **P < 0.01 vs corresponding untreated controls. **Figure S2.** Involvement of DNA methylation and Akt signaling pathway in FGF2 promoter regulation. Human coronary artery endothelial cells (HCAECs) were cotransfected with the reporter constructs -126/+179 or -126/+179 and phRL-TK (internal control), followed by incubation with L5 (100 μg/ml), 5-aza-dC (5-aza-deoxycytidine, 0.4 μg/ml), or Akt inhibitor (1 μg/ml) in the presence or absence of anti-MDA (0.15 μg/ml). Luciferase activity was expressed as a fold-increase of that for pGL3-basic. Values are expressed as the mean±SEM and are representative of 3 to 5 independent experiments. *P < 0.05 between the indicated pair. PBS, phosphate-buffered saline. **Figure S3.** Effects of oxLDL on DNA methyltransferase (DNMT) mRNA expression. Human coronary artery endothelial cells (HCAECs) were incubated with phosphate-buffered saline (PBS), native LDL (100 μg/ml), anti-MDA (0.15 μg/ml), oxLDL (100 μg/ml), or oxLDL+anti-MDA as indicated for 24 hours, and total RNA was subjected to real-time polymerase chain reaction analysis with specific primers for DNMT1, DNMT3A, DNMT3B, and β-actin. The values in the graph are expressed relative to that of the PBS control after normalization to β-actin and are presented as the mean ± SEM representative of 3 to 5 independent experiments. *P < 0.05 vs PBS-treated control; **P < 0.05 between the indicated pair (oxLDL+anti-MDA and oxLDL alone). **Table S1.** Primer sequences used for real-time polymerase chain reaction (PCR), reporter gene constructs, and CpG methylation studies. Click here for file
