# Supplementary material for: Malondialdehyde mediates oxidized LDL-induced coronary toxicity through the Akt-FGF2 pathway via DNA methylation
Source: J Biomed Sci. 2014 Feb 3;21(1):11. doi: 10.1186/1423-0127-21-11 (PMC3931320; doi:10.1186/1423-0127-21-11)
Supplement: Additional file 1: Figure S1 — Effects of oxLDL on DNA synthesis, cell viability, and cell death in cultured HCAECs. Cells were treated with increasing concentrations of oxLDL for 24 hours, and DNA synthesis (•), cell viability (□), and cell death (Δ) were assessed. Values are expressed as the mean±SEM (n=3). *P < 0.05, **P < 0.01 vs corresponding untreated controls. Figure S2. Involvement of DNA methylation and Akt signaling pathway in FGF2 promoter regulation. Human coronary artery endothelial cells (HCAECs) were cotransfected with the reporter constructs -126/+179 or -126/+179 and phRL-TK (internal control), followed by incubation with L5 (100 μg/ml), 5-aza-dC (5-aza-deoxycytidine, 0.4 μg/ml), or Akt inhibitor (1 μg/ml) in the presence or absence of anti-MDA (0.15 μg/ml). Luciferase activity was expressed as a fold-increase of that for pGL3-basic. Values are expressed as the mean±SEM and are representative of 3 to 5 independent experiments. *P < 0.05 between the indicated pair. PBS, phosphate-buffered saline. Figure S3. Effects of oxLDL on DNA methyltransferase (DNMT) mRNA expression. Human coronary artery endothelial cells (HCAECs) were incubated with phosphate-buffered saline (PBS), native LDL (100 μg/ml), anti-MDA (0.15 μg/ml), oxLDL (100 μg/ml), or oxLDL+anti-MDA as indicated for 24 hours, and total RNA was subjected to real-time polymerase chain reaction analysis with specific primers for DNMT1, DNMT3A, DNMT3B, and β-actin. The values in the graph are expressed relative to that of the PBS control after normalization to β-actin and are presented as the mean ± SEM representative of 3 to 5 independent experiments. *P < 0.05 vs PBS-treated control; **P < 0.05 between the indicated pair (oxLDL+anti-MDA and oxLDL alone). Table S1. Primer sequences used for real-time polymerase chain reaction (PCR), reporter gene constructs, and CpG methylation studies. [file 1423-0127-21-11-S1.pdf]

Malondialdehyde mediates oxidized LDL-induced coronary toxicity through the Akt-FGF2 pathway via DNA methylation

Tzu-Ching Yang, MS; Yi-Jie Chen, MS; Shwu-Fen Chang, PhD; Chu-Huang Chen, MD, PhD; Po-Yuan Chang, MD, PhD; Shao-Chun Lu,PhD

Online-Only Data Supplement

Methods

Real-time polymerase chain reaction (PCR)

Total RNA was isolated from human coronary artery endothelial cells (HCAECs) treated with phosphate-buffered saline (PBS), ox-LDL (100 μg/ml), or native (ie, control) low-density lipoprotein (LDL) (100 μg/ml) for 24 hours. Real-time PCR was performed by using total RNA and PCR primers for FGF2, DNMT1, DNMT3A, DNMT3B, or β-actin.[1,2] Briefly, total RNA (5 μg) in a 20-μl reaction mix was reverse-transcribed by using M-MLV Reverse Transcriptase (Promega, USA). Real-time PCR was performed in a total volume of 20 μl, including 10 μl of ABsolute QPCR SYBR Green Fluorescein Mix (ABgene, Epsom, United Kingdom), 1 μl of each primer at a 5 μmol/l concentration, and 1 μl of the reverse-transcribed cDNA template by using the iCycler iQ Real-Time PCR Detection System (BioRad, USA) with SYBR green according to the manufacturer’s protocol. The cycling conditions were as follows: 15 minutes at 95°C followed by 40 cycles of 10 seconds at 95°C and 45 seconds at 55°C. The fluorescence threshold value was calculated by using the iCycle iQ system software. The mRNA levels of each gene were normalized to those of β-actin.

Figure S1.

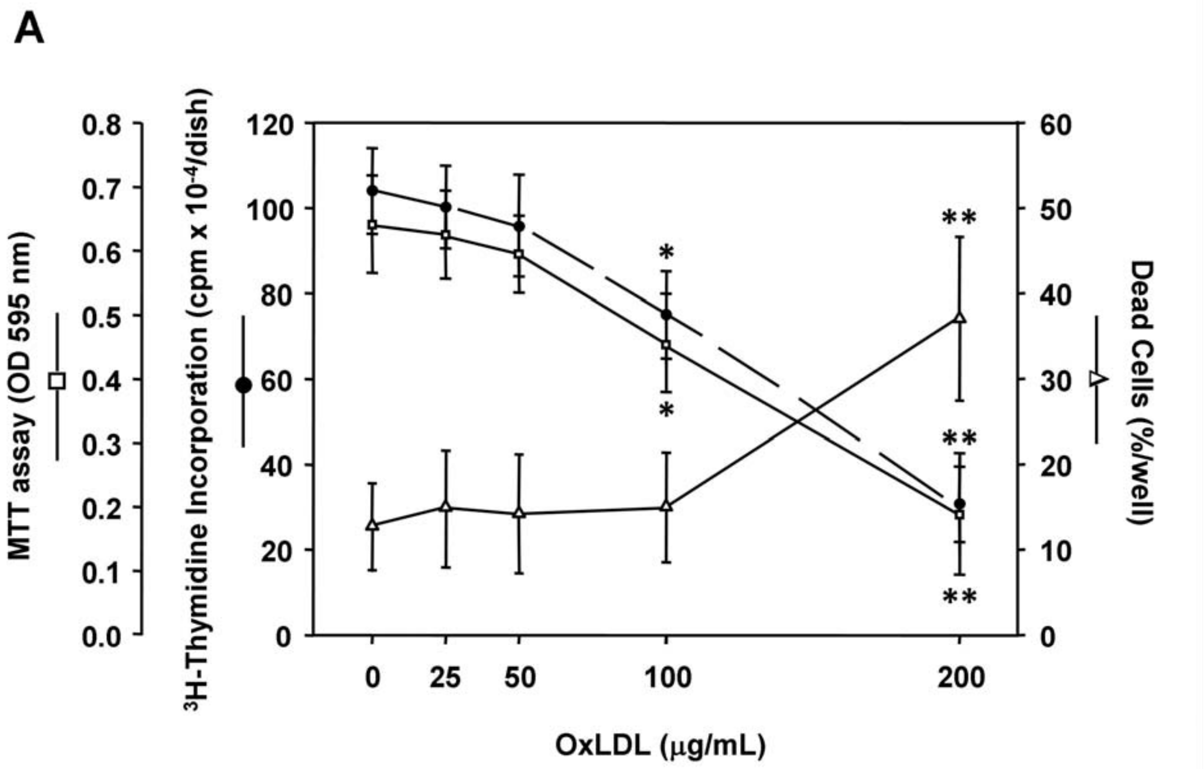

Figure S1. Effects of oxLDL on DNA synthesis, cell viability, and cell death in cultured HCAECs. Cells were treated with increasing concentrations of oxLDL for 24 hours, and DNA synthesis (•), cell viability (□), and cell death (Δ) were assessed. Values are expressed as the mean±SEM (n=3). **P*<0.05, ***P*<0.01 vs corresponding untreated controls.

Figure S2.

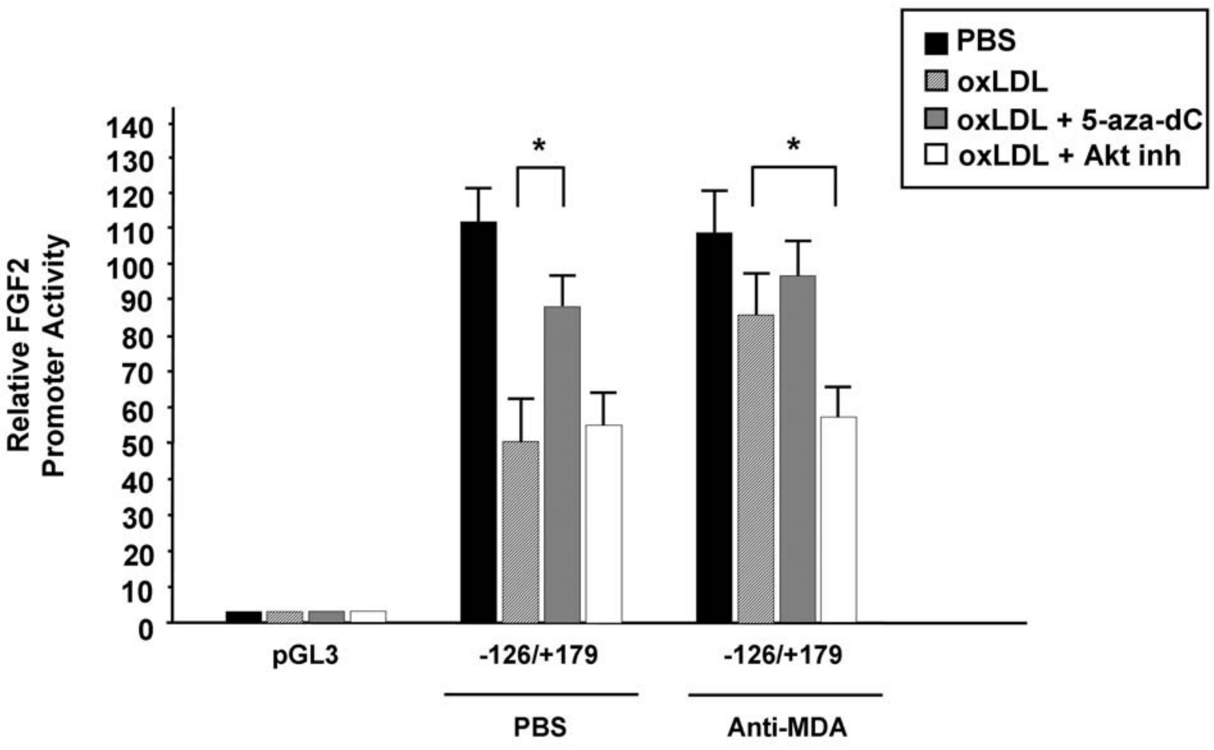

Figure S2. Involvement of DNA methylation and Akt signaling pathway in *FGF2* promoter regulation. Human coronary artery endothelial cells (HCAECs) were cotransfected with the reporter constructs -126/+179 or -126/+179 and phRL-TK (internal control), followed by incubation with L5 (100 μg/ml), 5-aza-dC (5-aza-deoxycytidine, 0.4 μg/ml), or Akt inhibitor (1 μg/ml) in the presence or absence of anti-MDA (0.15 μg/ml). Luciferase activity was expressed as a fold-increase of that for pGL3-basic. Values are expressed as the mean±SEM and are representative of 3 to 5 independent experiments. **P*<0.05 between the indicated pair. PBS, phosphate-buffered saline.

Figure S3.

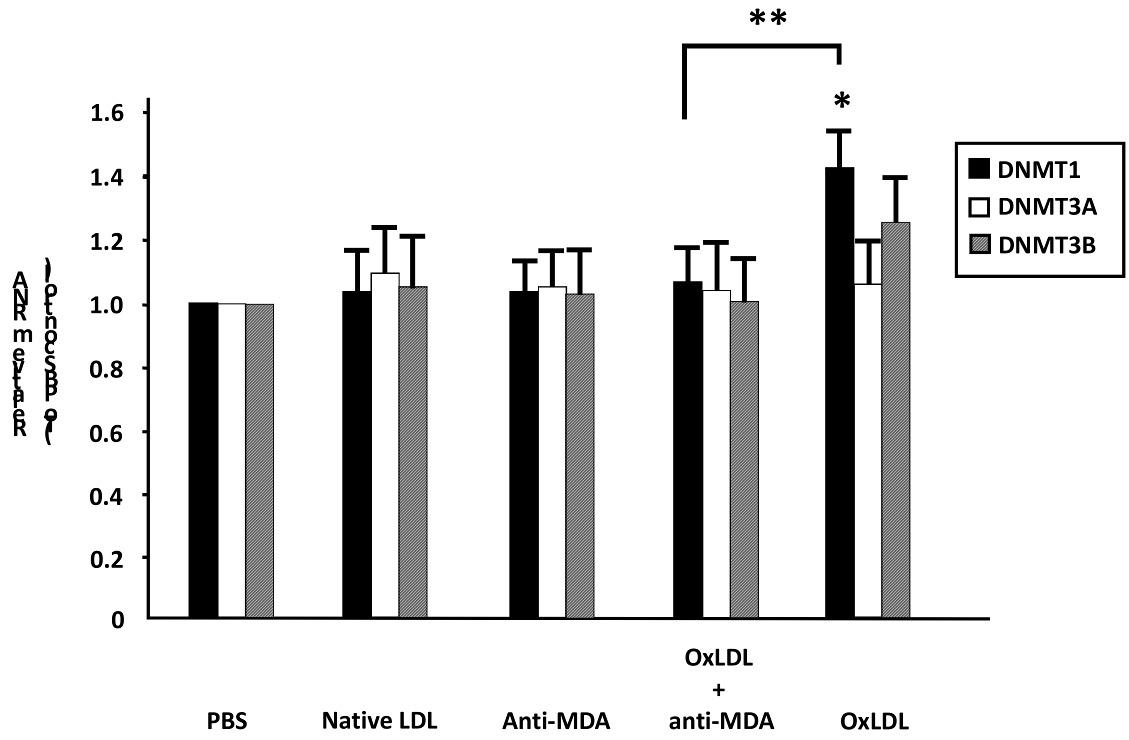

Figure S3. Effects of oxLDL on DNA methyltransferase (DNMT) mRNA expression. Human coronary artery endothelial cells (HCAECs) were incubated with phosphate-buffered saline (PBS), native LDL (100 μg/ml), anti-MDA (0.15 μg/ml), oxLDL (100 μg/ml), or oxLDL+anti-MDA as indicated for 24 hours, and total RNA was subjected to real-time polymerase chain reaction analysis with specific primers for *DNMT1*, *DNMT3A*, *DNMT3B*, and *β-actin*. The values in the graph are expressed relative to that of the PBS control after normalization to β-actin and are presented as the mean ± SEM representative of 3–5 independent experiments.. **P*<0.05 vs. PBS-treated control; ***P*<0.05 between the indicated pair (oxLDL+anti-MDA and oxLDL alone).

References

1. Chang PY, Luo S, Jiang T, Lee YT, Lu SC, Henry PD, Chen CH. Oxidized low-density lipoprotein down-regulates endothelial bFGF through a pertussis toxin–sensitive G-protein pathway: mediator role of platelet-activating factor–like phospholipids. *Circulation.* 2001;104:588-593

2. Chang PY, Chen YJ, Chang FH, Lu J, Huang WH, Yang TC, Lee YT, Chang SF, Lu SC, Chen CH. Aspirin protects human coronary artery endothelial cells against atherogenic electronegative LDL via an epigenetic mechanism: A novel cytoprotective role of aspirin in acute myocardial infarction. *Cardiovasc Res.* 2013;99:137-145

Table S1 Primer sequences used for real-time polymerase chain reaction (PCR), reporter gene constructs, and CpG methylation studies

	**Primer sequence (sense)**	**Primer sequence (antisense)**
**Real-time PCR**		
FGF2	5'-AACCGCGAGAAGATGACCCAGATCATGTTT-3'	5'-AGCAGCCGTGGCCATCTCTTGCTCGAAGTC-3'
DNMT1	5'-ACCGCTTCTACTTCCTCGAGGCCTA-3'	5'-GTTGCAGTCCTCTGTGAACACTGTGG-3'
DNMT3A	5'-CACACAGAAGCATATCCAGGAGTG-3'	5′-AGTGGACTGGGAAACCAAATACCC-3′
DNMT3B	5'-AATGTGAATCCAGCCAGGAAAGGC-3'	5′-ACTGGATTACACTCCAGGAACCG-3′
β-actin	5′-AACCGCGAGAAGATGACCCAGATCATGTTT-3′	5′-AGCAGCCGTGGCCATCTCTTGCTCGAAGTC-3′
**Reporter gene constructs[a]**		
–126 to +43	5′-ACGCGTCAGGGAATGCCAAAGCCCTGC-3′	5′-CTCGAGCTACTCGCTCGGGTTTTCTGGGGC-3′
–126 to +179	5′-ACGCGTCAGGGAATGCCAAAGCCCTGC-3′	5′-CTCGAGGATCCCGTTGCAACCGCGGGCA-3′

+24 to +179	5′-ACGCGTCAGAAAACCCGAGCGAGTAG-3′	5′-CTCGAGGATCCCGTTGCAACCGCGGGCA-3′
**DNA methylation studies**		
CpG Primer 1	5′-TTGGATTGTAATTTTTTTATTTTGG-3′	5′-AACTTTAACATTCCCTAAACTCCAC-3′
CpG Primer 2	5′-GTGGAGTTTAGGGAATGTTAAAGTT-3′	5′-ATTTTCTAAAACCAACCTCTAAATT-3′

[a]Restriction sites added for cloning were *Mlu-1* (underlined) and *Xho-1* (double underlined). FGF2, fibroblast growth factor-2; DNMT, DNA methyltransferase.
